# Single-Cell Lipidomics Using Analytical Flow LC-MS Characterizes the Response to Chemotherapy in Cultured Pancreatic Cancer Cells

**DOI:** 10.1021/acs.analchem.3c02854

**Published:** 2023-09-19

**Authors:** Kyle D. G. Saunders, Johanna von Gerichten, Holly-May Lewis, Priyanka Gupta, Matt Spick, Catia Costa, Eirini Velliou, Melanie J. Bailey

**Affiliations:** † Department of Chemistry, University of Surrey, Guildford GU2 7XH, U.K.; ‡ Faculty of Health & Medical Sciences, University of Surrey, Guildford GU2 7XH, U.K.; § Centre for 3D Models of Health and Disease, University College London—Division of Surgery and Interventional Science, London W1W 7TY, U.K.; ∥ Ion Beam Centre, University of Surrey, Guildford GU2 7XH, U.K.

## Abstract

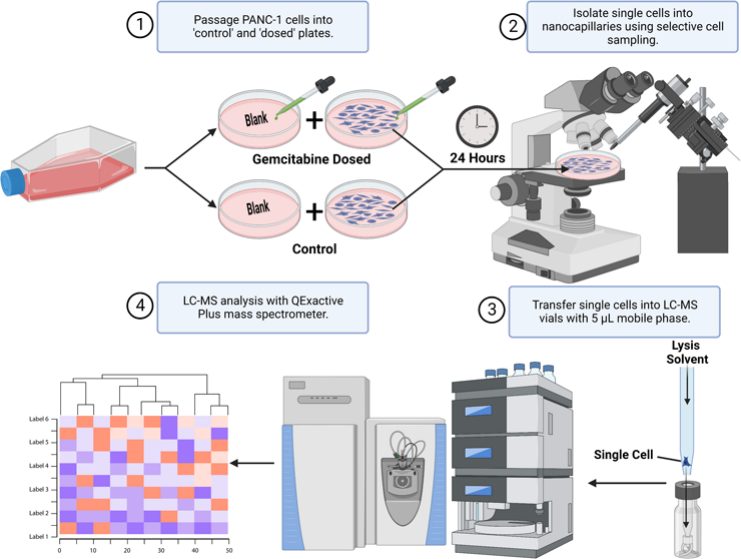

In this work, we demonstrate the development and first application of nanocapillary sampling followed by analytical flow liquid chromatography–mass spectrometry for single-cell lipidomics. Around 260 lipids were tentatively identified in a single cell, demonstrating remarkable sensitivity. Human pancreatic ductal adenocarcinoma cells (PANC-1) treated with the chemotherapeutic drug gemcitabine can be distinguished from controls solely on the basis of their single-cell lipid profiles. Notably, the relative abundance of LPC(0:0/16:0) was significantly affected in gemcitabine-treated cells, in agreement with previous work in bulk. This work serves as a proof of concept that live cells can be sampled selectively and then characterized using automated and widely available analytical workflows, providing biologically relevant outputs.

## Introduction

Lipidomics is a powerful tool for the analysis of biological systems, providing insights into cellular physiology, disease progression, and drug discovery. Typically, lipidomics is performed on bulk tissue or large cell populations, which fails to capture cellular heterogeneity and may result in an incomplete understanding of metabolic processes.^1^ Liquid chromatography–tandem mass spectrometry (LC-MS/MS) is the primary tool for lipidomics due to its high sensitivity and depth of information. Various systems for cell isolation have been developed and can be coupled with LC-MS/MS, for example, in single-cell proteomics,^2,3^ but to our knowledge, untargeted LC-MS/MS lipidomics analysis on single cells has not yet been demonstrated.

Mass spectrometry imaging (MSI) methods such as matrix-assisted laser desorption/ionization (MALDI) and secondary ion mass spectrometry (SIMS) have been used to rapidly detect metabolites and lipids in single cells.^4−9^ However, unlike LC-MS, MSI techniques do not have any separation of compounds prior to ionization, meaning that ionization suppression reduces sensitivity and precision.^10^ Furthermore, MSI techniques have not demonstrated tandem mass spectrometry (MS/MS) directly on single cells in order to assign more confident identifications. Instead, peaks are assigned by database matching, or a bulk extract is analyzed in parallel by LC-MS/MS to confirm peak assignment.^11^ Finally, MSI is incompatible with live cell analysis because ions cannot be extracted from cells growing in media.^12,13^

Capillary sampling under microscope observation followed by nanoelectrospray ionization (NSI) is a technique that has been employed for the detection of metabolites and lipids in single living cells.^14−17^ This approach is advantageous because it can isolate live single cells in their native state, and spatial information is retained through using microscopy.^18,19^ However, NSI suffers from challenges with precision^20^ and lacks automation, both in data acquisition and analysis.

Chromatography can mitigate ionization suppression and matrix effects seen in mass spectra, as well as add another level of identification through retention time. We have previously demonstrated that nanocapillary sampling coupled to LC-MS is capable of detecting and quantifying drug compounds in single cells, as well as tentatively identifying some lipid features.^21^ This, therefore, initiated the development of a single-cell lipidomics approach focusing on coverage and identification. The ability to sample living cells and then analyze their lipidomic profiles with LC-MS/MS could allow insight into a vast number of biological applications.

A particular challenge in oncological research is pancreatic ductal adenocarcinoma (PDAC). PDAC is one of the most deadly forms of cancer, with a 5-year survival rate of less than 10%.^22^ Gemcitabine is considered the first choice for chemotherapeutic treatment of PDAC but nevertheless has a very poor response rate.^23^ Previous research has investigated the lipid response of PDAC models to gemcitabine, giving insight into treatment resistance in cancer,^24−27^ so this serves as an interesting biological system by which to test the single-cell lipidomics approach developed here.

This work is the first demonstration of single-cell nanocapillary sampling coupled to analytical flow LC-MS lipidomics. The modifications required to adapt the previous methodology for drug analysis into a fit for purpose lipidomics workflow are described. We have optimized the transfer of lipids from capillaries into LC vials, the carrier volume of solvent, the chromatographic separation, and mass spectrometry parameters, specifically focusing on low volume, dilute samples. We have developed a strategy for peak annotation based on the assignment of low-confidence (*m*/*z* value, passed noise filtration), medium-confidence (database matched via MS1 peak and manually curated), and high-confidence (MS2 data) features. The method provides the further benefit of being able to separate and characterize isobars, which is inaccessible to mass spectrometry imaging. The significance of this work is that the method has the ability to recognize a putative response to biological stimuli based solely on the lipidomic profile of single cells. This work will be of interest to researchers from various backgrounds paying attention to the current state of single-cell omics using LC-MS. We will also address the key challenges, as well as the potential for answering critical biological questions.

## Methods and Materials

### Chemicals and Reagents

A United States Pharmacopeia reference standard of gemcitabine hydrochloride (200 mg, catalog No. 1288463) was obtained from Sigma-Aldrich. A deuterated lipid standard mix EquiSPLASH (Avanti Polar Lipids, catalog No. 330731) was also purchased from Sigma-Aldrich for use as a multiclass internal standard, described in Table S1. A lysate of human cervical cancer (HeLa) cells was purchased from Caltag Medsystems (Buckingham, U.K., 200 μg catalog No. L013 V2) for use as a single-cell adjacent standard. Chromatography solvents methanol (MeOH), ethanol (EtOH), isopropyl alcohol (IPA), acetonitrile (ACN), and water were Optima LC-MS grade and purchased from Fisher Scientific. Chloroform used for lipid extraction was high-performance liquid chromatography (HPLC) grade (99.5+%) and also purchased from Fisher Scientific. Dulbecco’s phosphate-buffered saline (DPBS) was purchased from Sigma-Aldrich (catalog No. D8537). The cell culture media was prepared, as described by Wishart et al.^25,28−30^ More specifically, Dulbecco’s modified Eagle’s medium (DMEM) with high glucose (Sigma-Aldrich, Merck, U.K., catalog No. 21969035) was supplemented with 10% fetal bovine serum (Fisher Scientific, U.K., catalog No. 11550356), 1% penicillin/streptomycin (Fisher Scientific, U.K., catalog No. 15140122), and 2 mM l-glutamine (Sigma-Aldrich, Merck, U.K., catalog No. 25030024).

### Cell Culture and Gemcitabine Treatment

Human pancreatic adenocarcinoma cells (PANC-1, Merck, U.K.) were used for single-cell and cell extract measurements. Cells were cultured in Corning T25 culture flasks (Merck, U.K.) in DMEM prepared as described above. During culturing, the cells were kept in a cell culture incubator at 37 °C with 21% O_2_ and 5% CO_2_. Cell culture media was replaced on alternating days, and cells were passaged approximately once a week, when confluency reached 80–90%. Prior to single-cell sampling, approximately 200,000 cells each were seeded into a fresh T25 flask and BioLite cell culture treated dishes (Fisher Scientific, U.K., catalog No. 11844335). The same volume of cell culture media (containing no cells) was simultaneously aliquoted into cell culture dishes to serve as the representative blanks.

Once cells had been cultured for 24 h, the media was replaced with an aliquot supplemented with 10 μM gemcitabine hydrochloride, a nucleoside chemotherapy drug commonly used in pancreatic cancer treatment.^31^ The media of the control cells was simply replaced with drug-free media. Both groups, control and treated, were left to incubate for a further 24 h before sampling. Culture media were replaced with DPBS for the duration of sampling.

### Lipid Extract from Bulk Cells

A lipid extraction was performed on a population of PANC-1 cells grown to approximately 90% confluency. Cells were trypsinized and counted with a hemocytometer before being pelleted by centrifugation at 150*g* and washed with ice-cold DPBS. The cell pellet was suspended in 1 mL of water and flash frozen in liquid nitrogen. The cell pellet was then subjected to two cycles of freeze–thaw (37 °C for 10 min, liquid nitrogen for 30 s) to aid cell lysis. Lipids were extracted by a modified Folch extraction according to the protocol described by Zhang et al. using a chilled solution of methanol/chloroform (1:2 v/v) supplemented with 0.01% butylated hydroxytoluene (BHT) to prevent lipid oxidation.^32,33^ The bottom layer of the extract was taken and dried down under nitrogen gas, stored at −80 °C, and reconstituted on the day of analysis in the starting mobile phase of the C30 chromatographic method described in Table S2. The lipid extract was diluted to a concentration of 2800 cells/μL.

### Single-Cell Sampling

Single cells from the control and gemcitabine-treated culture dishes were collected by nanocapillary sampling into borosilicate nanocapillaries, created to the specification using a PUL-1000 tip puller (World Precision Instruments). Capillaries containing a single cell were stored at −80 °C until the day of analysis. Nanocapillary sampling was controlled using a nanomanipulator (Attocube, Germany), which allows for user-guided movement in three dimensions with fine motor control. The nanocapillary was guided to the surface, and a pressure injector (PM1000 microinjector, MicroData Instrument) was used to aspirate the cell into the capillary, as shown in Figure S1.

### Sample Transfer

To perform LC-MS analysis on single cells, cells must be transferred from the nanocapillary within which they are isolated to a vial suitable for handling low sample volumes. This was achieved by backfilling nanocapillaries with 5 μL lysis solvent (starting mobile phase composition spiked with EquiSPLASH as an internal standard, 16 ng/mL, see Table S2) and using a gas syringe with a Luer lock adapter using a syringe pump to elute the solution into a vial at a flow rate of 65 μL/min (Figure S2).

### Characterization of Transfer Efficiency

#### LC Workflow

EquiSPLASH (16 ng/mL in 50:50 MeOH/EtOH) was used to evaluate the efficiency of transferring the low sample volumes required for single-cell analysis. To assess the transfer efficiency of the LC system, 5 μL of the standard solution was aliquoted directly into 5 QSert LC vials with a 300 μL insert (Sigma-Aldrich, U.K., catalog No. 29391-U). To assess the efficiency with a larger carrier volume, 5 μL of the solution was aliquoted directly into five additional vials and diluted to a total volume of 15 μL with 50:50 MeOH/EtOH. The injection volume was increased to 15 μL for these samples so as to maintain the same mass loaded onto the column. For comparison, 200 μL of the standard solution was aliquoted in a separate vial, and five replicate injections of 5 μL were taken from this vial to serve as a control. Samples were analyzed by LC-MS using the C18-based gradient (Table S2), and transfer efficiency was determined using eq S1, found in the Supporting Information.

#### Capillary to Column

To assess the efficiency of transferring lipid samples out of nanocapillaries, 5 μL of the standard solution was backfilled into five empty nanocapillaries, and the solution was pushed out into LC vials using the gas syringe apparatus described above. Another five nanocapillaries were backfilled, and the contents were eluted into LC vials, but the final volume was made up to 15 μL using 50:50 MeOH/EtOH in order to explore the effect of a larger carrier volume. The same control, gradient, and equation for determination of transfer efficiency as the LC workflow analysis was used as described above.

#### Liquid Chromatography

A dilution of the bulk lipid extraction from PANC-1 cells (70 cells/μL) was analyzed with a C18 chromatography column as described previously,^21^ and with a C30 chromatography column based on Narváez–Rivas to compare the lipid coverage achieved by the two gradients using a small number of cells.^34^ The parameters of both gradients are described in Table S2. Consequently, single cells were analyzed using the C30-based LC-MS gradient. To obtain fragmentation data, a lipid extraction of bulk cells (2800 cells/μL) was analyzed in parallel to the single cells using a data-dependent acquisition (DDA) MS/MS method (parameters in Table S3).

#### Mass Spectrometry Parameters

Cells, cell lipid extracts, and standards were analyzed using a Thermo Fisher Scientific (Massachusetts) Ultimate 3000 UHPLC system coupled to a Thermo Fisher Scientific Q-Exactive Plus Orbitrap mass spectrometer. Unless otherwise stated, the ionization source was a heated electrospray ionization (HESI) probe set to 320 °C, automatic gain control (AGC) with a target of 1 × 10^6^, HESI probe spray voltage of 4 kV, and mass range *m*/*z* 200–1400 with a resolution of 70,000.

To improve sensitivity to lipids at the single-cell level, optimization of the capillary temperature of the electrospray probe and automatic gain control (AGC) target was carried out. Capillary temperatures between 280 and 380 °C were investigated in 20 °C intervals using EquiSPLASH (16 ng/mL in 50:50 methanol/ethanol). AGC settings were assessed with a maximum ion injection time of 400 ms to allow all injections to reach the target. A lysate of HeLa cells was used for this experiment, diluted with mobile phase (starting composition) of the C30 chromatography method (Table S2) to single-cell level (1 cell/μL, 5 μL injection).

#### MS/MS and Sensitivity

To assess the detection limit of the instrument during MS/MS acquisition, the lipid extract from bulk cells was diluted in a series ranging from 2800 to 9 cells/μL. Five microliter injections were used for the LC-MS/MS gradient, therefore giving a range of 14,000–45 cells/injection. A DDA MS/MS method was used to obtain fragmentation data on the lipid extraction with a *m*/*z* exclusion list (Table S3). This exclusion list was generated from five replicate blank injections (mobile phase).

#### Data Processing

Lipostar 2 (Molecular Discovery, Italy) was used for data processing. Data were subjected to a 3× signal/noise ratio filter (based on mass spectrum signal intensity) before lipid identification in the software. Gap filling was not used for single-cell data due to the inherent heterogeneity of single cells. Blank subtraction, 3× signal/noise filtering (based on peak area), normalization to the lipid class-matched internal standard, EquiSPLASH, and curation of identifications were then processed manually outside of the software using Excel (Microsoft) and Freestyle (Thermo Scientific). Only lipid identifications belonging to classes detectable in the internal standard (10 classes, namely, PC, PE, PS, PG, LPC, LPE, TG, DG, SM, and Cer) were retained for analysis. Multivariate analysis was then carried out using a MetaboAnalyst (Canada). Furthermore, to effectively compare gemcitabine-treated cells and the controls, additional filtering was applied to the data preprocessing techniques described above. Lipid identifications made in less than 60% of either the control or gemcitabine cell samples, or without a defined chromatography peak, were discarded.

Identification of lipids at the single-cell level is present in three levels of confidence defined in this work. “Low-confidence” represents features with an *m*/*z* value that has been detected and successfully passed signal/noise filtration and blank subtraction. “Medium-confidence” identifications are features that have passed preprocessing steps, have a named match in the LipidMAPS database (10 ppm tolerance), and are represented in the lipid classes observed in the EquiSPLASH internal standard. However, no fragmentation data are available to confirm the identification at the medium-confidence level. “High-confidence” features have database matches to the *m*/*z* value as well as fragmentation spectra matched from the bulk.

## Results and Discussion

The present findings show that lipidomic analysis of single cells using standard analytical liquid chromatography coupled to mass spectrometry can generate research-relevant outputs, demonstrated for the cancer treatment drug gemcitabine on pancreatic cancer cells.

**Figure 1:**
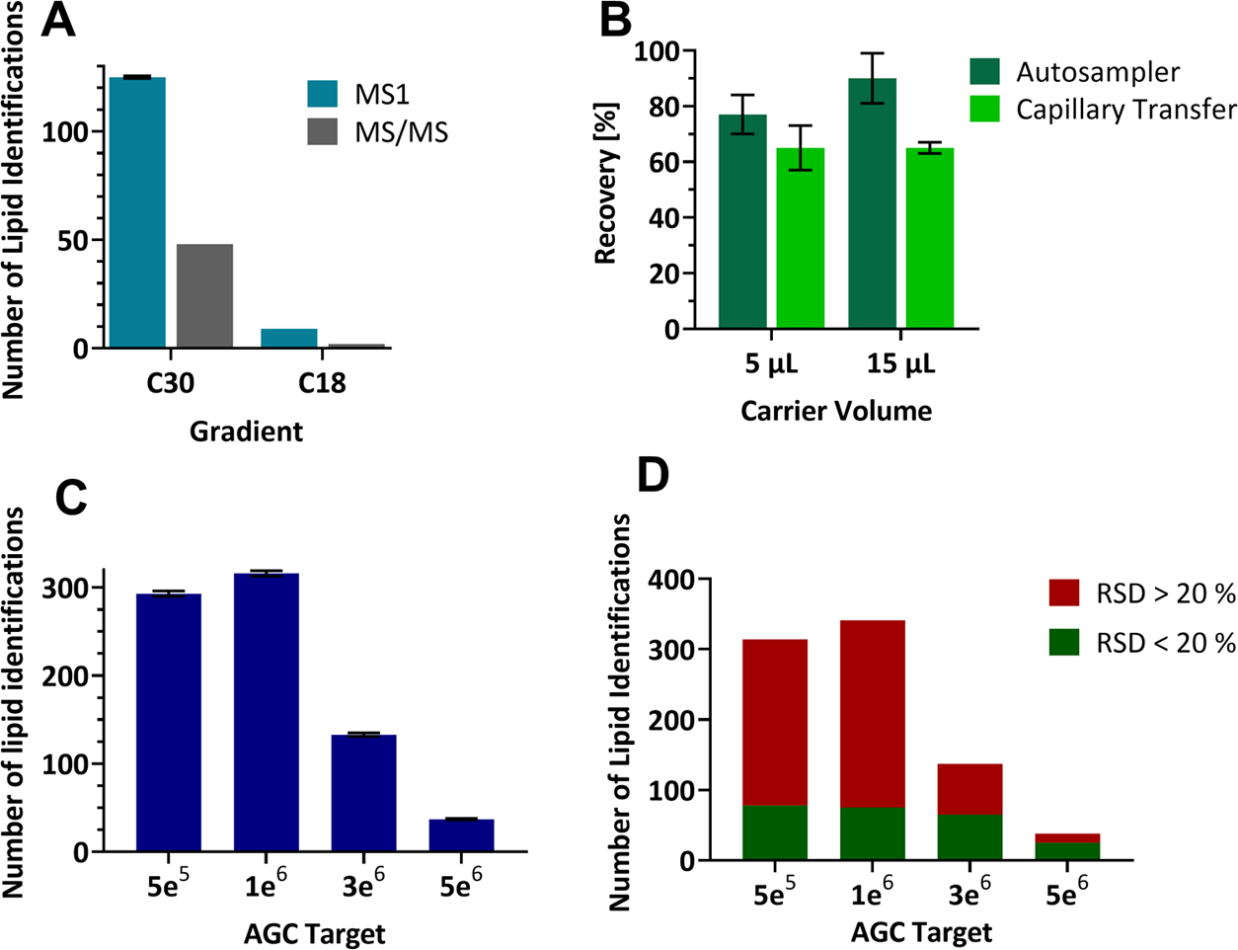
(A) Number of tentative and MS/MS-confirmed lipid identifications made in the 70 cells/μL lipid extract between C30- and C18-based chromatographic methods. (B) Average transfer efficiency of aliquots of 16 ng/mL lipid standards using two volumes of carrier solvent. (C) Analysis of HeLa lysate diluted to a single-cell concentration demonstrated the number of tentative identifications observed with increasing AGC in triplicate injections. (D) Lipid identifications were proportioned by their relative standard deviation (RSD) with increasing AGC target. All error bars are 1 standard deviation (SD).

The results of optimizing chromatography, sample transfer efficiency, and automatic gain control are shown in Figure 1.

### Chromatography

The number of lipid features in dilute lipid extract for both gradients (tentative and MS/MS-confirmed) is shown in Figure 1A. The number of identified lipids in dilute bulk extract based on the C30 chromatography column is 14 and 24-fold higher for tentative and MS/MS-confirmed ID’s, respectively. The lipid class separation was significantly improved with the C30 chromatography column when comparing EquiSPLASH standards. Chromatograms for PC (15:0/18:1(d7)) and PE (15:0/18:1(d7)) are shown in Figure S3A,B, respectively, demonstrating an increase in the resolution by 26% for these two lipid standards.

We clearly demonstrate that the number of lipids identified (tentatively or with matched MS/MS fragments) in dilute samples is significantly improved by using the longer, C30-based chromatography column and gradient adapted from Narváez–Rivas et al.^34^ as opposed to the C18-based chromatography column and shorter gradient used in previous single-cell work.^21^ This improvement in lipid coverage was observed despite the faster flow rate of the C30 gradient, which should result in greater sample dilution and therefore reduced sensitivity. The more extensive separation (from *R* = 1.8 to 2.3 for PC-PE; see Figure S3A,B) likely results in fewer lipid species coeluting, reducing ionization suppression. The C30-based gradient was, therefore, adopted for single-cell work. While these results demonstrate a significant improvement in sensitivity, a more detailed optimization of the chromatographic method was not explored. This highlights the promising opportunity for future LC optimization to make further gains in sensitivity by enhancing separation of single-cell lipids. One such area of investigation for single-cell analysis is micro- and nanoflow LC-MS, as they both offer improved sensitivity and ionization efficiency relative to the flow rates used in this work due to reduced sample dilution in the column.^35^

### Mass Spectrometry Parameters

The impact of electrospray probe temperature on lipid class intensity is shown in the Supporting Information (Figures S4 and S5). The optimal temperature for ionization ranged from 280 to 380 °C based on the class in question (Table S4). Therefore, a temperature of 320 °C (the average of each optimal temperature) was found to be a suitable compromise for the single-cell work.

The number of tentative (medium-confidence, as described above) lipid identifications detected in single-cell dilution HeLa lysate with increasing AGC targets is demonstrated in Figure 1C, while the number of identifications made with acceptable precision (RSD < 20%) is demonstrated in Figure 1D. An AGC target of 1 × 10^6^ showed the highest total number of identified lipids, followed by an AGC target of 5 × 10^5^ (*p* < 0.01). The number of lipid features tentatively identified at single-cell concentrations appears to be greatly affected by the automatic gain control target set for data acquisition. For proteomics, a high AGC target is a valid approach for single-cell work as more of the available ion pool will be analyzed, conferring greater signal/noise ratios and repeatability of signals.^36^ However, increasing the AGC/IIT increases the duty cycle, which can reduce the coverage of the analytes observed. In this work, the same observations have been made for lipids, whereby increasing the AGC beyond 1 × 10^6^ significantly reduces the number of lipid features. Figure 1D effectively demonstrates the effect on signal repeatability, as the proportion of lipid identifications with acceptable precision (relative standard deviation <20%) is greatly improved with the increased AGC target. However, although the precision is improved proportionally, the total number of lipids identified with acceptable precision is higher for AGC targets of 5 × 10^5^ and 1 × 10^6^. In light of these findings, an AGC target of 1 × 10^6^ was adopted in the proceeding work to maximize the number of tentative identifications and still obtain a suitable number of features with acceptable precision in single cells.

### Transfer Efficiency

The transfer efficiency of the LC autosampler and capillary is demonstrated in Figure 1B for 5 and 15 μL of carrier solvent. An *F*-test between the capillary transfer for 5 and 15 μL (*p* < 0.01) reveals a significant decrease in the variance when the larger carrier volume is used. We have previously evaluated the transfer efficiency of drug compounds using a hand-held gas syringe to push the contents of nanocapillaries into an LC vial.^21^ Transfer efficiencies of 70–100% for several drug compounds were observed, but this method did not reproduce well for lipid standards (data not shown). This work reports a new transfer approach, described in the Methods and Materials section, to transfer multiple lipid classes.

First, considering the transfer efficiency of the LC workflow, there appears to be significant sample loss with a carrier volume of 5 μL across the lipid classes compared to 15 μL (*p* < 0.001), possibly due to solvent evaporation and the inability of the autosampler to take up the entirety of the volume from the vials. Diluting the sample to 15 μL and increasing the injection volume (thereby introducing the same mass of sample as in the control) confers a significant improvement in transfer efficiency across all lipid classes. However, a greater sample loss was observed when the standard had been transferred through the nanocapillary (*p* < 0.001 at both 5 and 15 μL of carrier volume). This is possibly due to the deposition of lipids on the inside of the glass capillary and evaporation of the solvent when using the gas syringe. The transfer efficiency observed in this work still falls within the range observed previously for drug compounds (70–100%).^21^ There is no significant difference in the transfer efficiency at 5 or 15 μL final volume from the capillary; the limitation on transfer is therefore between capillary and LC vial. Due to the decreased variability in recovery, a carrier volume of 15 μL was adopted for proceeding single-cell work.

These results remain only analogous to the transfer of single-cell lipids from the capillary to the detector. The lipid standards are homogeneous in solution, whereas most cellular lipids are bound up in membranes and organelles before lysis in the capillary. While this experiment cannot account for the efficiency of cell lysis and heterogeneity of the sample material, it still evidences that the transfer of lipids in this workflow suffers from sample loss and that work is needed in the near future to improve the transfer of sample material. A suggestion for future investigation is transfer of the whole cell from the sampling capillary to the vial or well plate before lysis, in order to avoid deposition of lipids inside the capillary. This could, in theory, be achieved with microfluidics or robotics. One solution is the use of newly commercialized platforms capable of single-cell sampling and deposition of intact cells, which can sample using gentle capillary forces and confirm deposition of the live cell through microscopy.^37,38^ This prelysis cell transfer was not attempted in this work as it could possibly present increased mechanical and metabolic stress to the cell before the metabolism is quenched by lysis. This would need to be characterized and addressed by future work looking to transfer whole cells to LC vials. Furthermore, gains might be made through investigation of LC vials and capillaries with a variety of coatings in order to minimize surface adherence of lipids. The LC autosampler would then remain the limiting step for sample transfer, which this work demonstrates to be approximately 90% for most of the observed lipid classes with an increased carrier volume.

### MS/MS Coverage and Sensitivity

**Figure 2:**
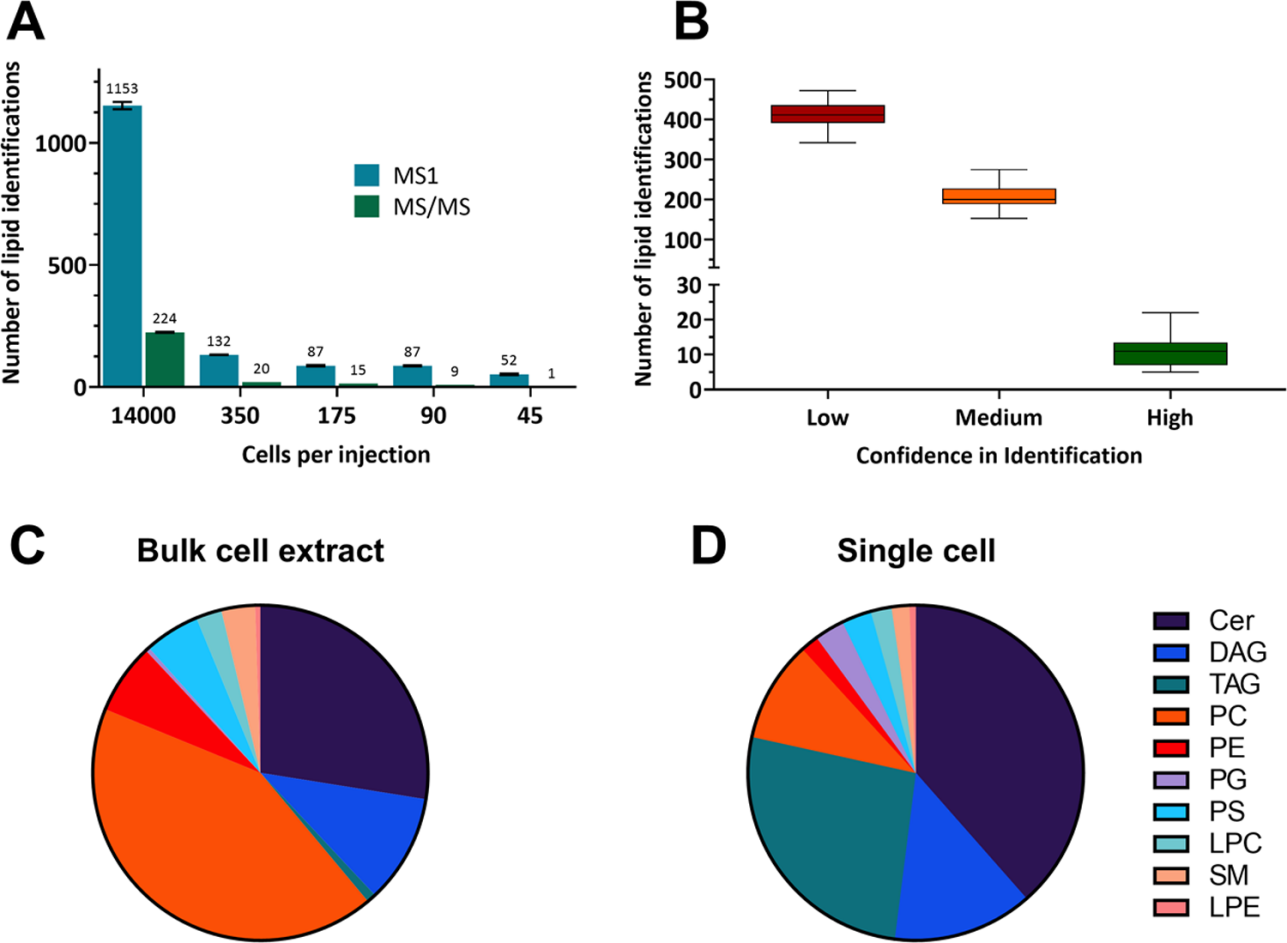
(A) Tentative and MS/MS-confirmed lipid identifications in a dilution series of PANC-1 lipid extraction using the DDA method. Error bars = 1 SD. (B) Lipid identifications in 14 single cells, split by their confidence. (C) Composition of internal standard matched lipids identified with at least medium-confidence as defined above in a bulk lipid extraction of PANC-1 cells. (D) Composition of standard matched lipids was identified in 14 single cells.

The number of lipids identified both tentatively (medium-confidence) and with MS/MS confirmation (high-confidence) using a DDA MS/MS method in the lipid extraction dilution series is shown in Figure 2A. The number of lipids identified with DDA MS/MS ranges from 224 to 1 over the cell dilution, showing that already, with the first dilution of 350 cells, only 20 lipids are identified by DDA MS/MS. To gain high-confidence lipid identifications, tandem mass spectrometry data is always required so that fragments can be matched to reference spectra. The lipid extraction of bulk cells demonstrates a number of MS/MS-confirmed lipid identifications in the same range as observed by Zhang et al. using a similar extraction protocol.^32^ However, when the extract is diluted to a low cell concentration, the number of identifications is severely reduced.

At low cell concentrations, the ability to confirm lipid identifications with MS/MS fragmentation is clearly limited by using this workflow. Even with 45 cells, the DDA method used was unable to sufficiently identify more than one lipid with MS/MS confirmation. These data suggest that a DDA MS/MS experiment performed directly on single-cell samples lacks the required sensitivity for detailed analysis and that alternative means of obtaining fragmentation data must be used. For MSI techniques used for single-cell analysis such as matrix-assisted laser desorption ionization mass spectrometry (MALDI-MS), obtaining MS/MS data directly from the single cell is also challenging.^11^ A growing trend in single-cell MALDI is parallel LC-MS/MS analysis of pooled cellular materials to match the observed *m*/*z* values in the single-cell data. full scan MS analysis of single cells with parallel DDA MS/MS analysis of a lipid extraction of bulk cells was adopted in proceeding single-cell work in order to obtain MS/MS validation of lipid identifications.

### Lipid Identifications in Single Cells

The number of lipid identifications in 14 single cells (analyzed by full scan) and their level of confidence are shown in Figure 2B. Across all levels of confidence, the total number of identifications ranged from 552 to 758 lipids. An average of 220 medium/high-confidence lipid identifications were obtained per single cell. Obtaining fragmentation data from a bulk sample in parallel results in significantly more identifications than directly acquiring spectra on the dilute cell material (Figure 2A). However, the number of high-confidence MS/MS-confirmed identifications still represents a small proportion of the total number of lipid identifications. The reason for this disparity is unknown, but a likely explanation is the difference in lipid extraction protocols and matrix effects between the two sample types. Supporting this notion, there are some lipids which appear in single-cell samples that do not appear in the bulk. Figures S6 and S7 demonstrate two lipid features that both have very large responses at the bulk level and are also detected in single cells. Figure S8, on the other hand, demonstrates that Cer(34:0) does not appear in the bulk despite appearing in single cells. This is possibly due to decreased ionization suppression from more abundant lipids that coelute in a bulk concentration.

Although database matching is widely used in imaging mass spectrometry, for meaningful biological interpretation, the lipids reported in omics work must have putative identifications.^39^ These observations highlight the need to optimize confidence in lipid identification at the single-cell level in future work. The method demonstrated here provides *some* high-confidence identifications, which likely are biased toward higher abundance lipids. However, a major appeal of single-cell analysis is the ability to detect signals that may be missed at the bulk level as they are expressed by a small number of cells. Consequently, the goal should be acquisition of fragmentation data directly from the single cell. There are several ways to address this issue, such as improving the duty cycle with faster instrumentation or taking advantage in recent improvements in ion trapping and lossless ion transfer.^40−44^

### Lipid Composition—Single Cell vs Bulk Cells

The identified lipid compositions of the bulk extraction and single cells (Figure 2C,D, respectively) are presented as the sum of individual lipids into the respective lipid class. The largest difference observed was the proportion of PCs identified between single cells (9.8%) and bulk cell extraction (42.3%). Overall, there is some consistency between the observed classes of lipids in single cells and bulk extracts; however, the disparity between PCs and TAGs is considerable (for TAGs, 26.4% in single cells, 0.9% in bulk). One explanation is that the single cells selected had an abundance of TAG containing lipid droplets relative to the bulk population.^45^ The observation of increased DAGs is shown and supports this notion (13.8% in single cells, 10.5% in bulk), although the increase is not as large as TAGs. An alternative explanation is that the extraction method is responsible for the differences. The lipid extraction of bulk cells was performed with methanol/chloroform, whereas the single cells were lysed using the starting mobile phase, primarily isopropyl alcohol and acetonitrile. (Single cells were lysed in this way to prevent further evaporation at low sample volumes and to maintain compatibility with the LC-MS method.) Alternatively, a third explanation is the difference in matrix effects between the very dilute samples and the more concentrated lipid extraction.

### Gemcitabine Treatment and Single-Cell Analysis

**Figure 3:**
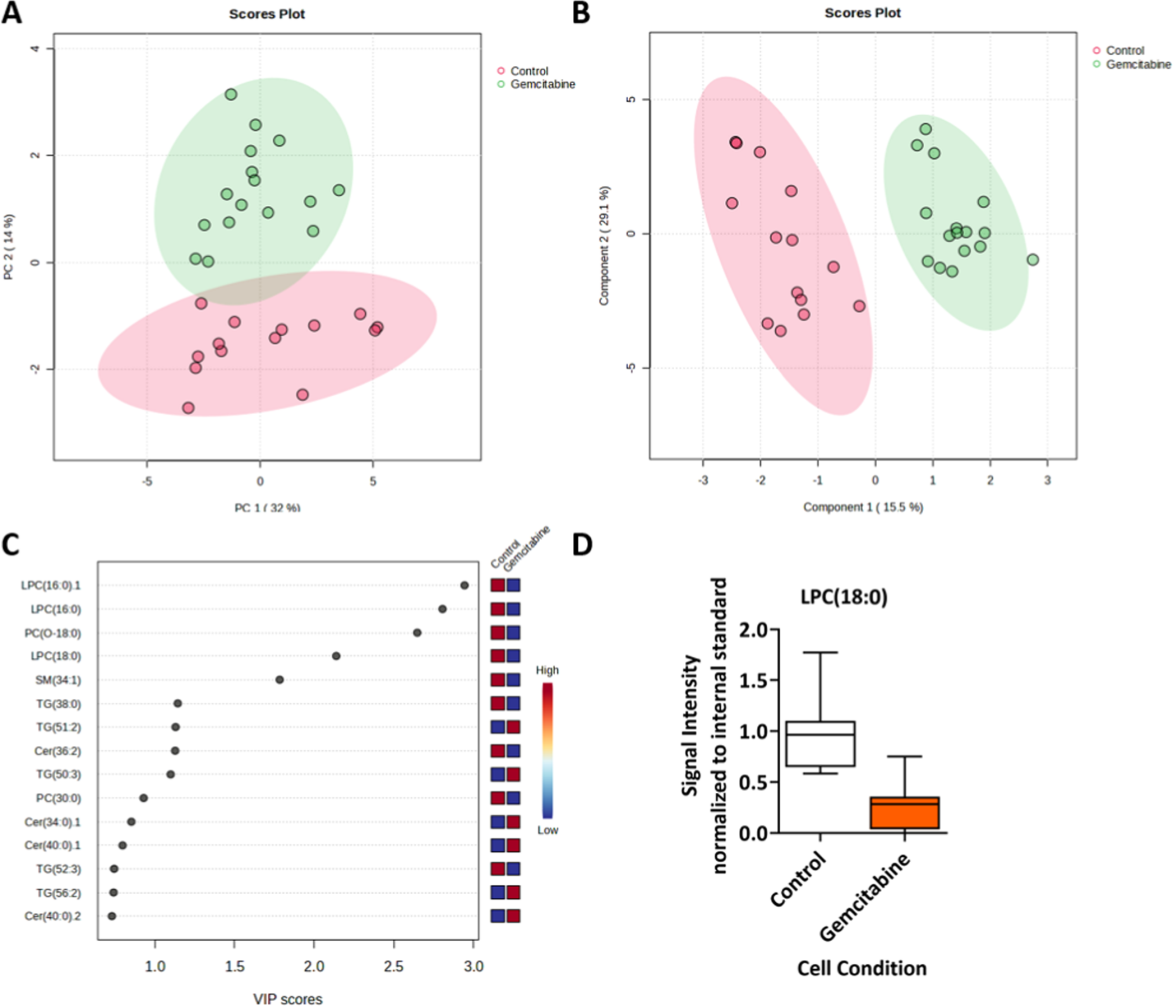
(A) PCA of the lipid profiles of control cells (*n* = 14) and gemcitabine-treated cells (*n* = 16). (B) PLS-DA spectra of the lipid profiles from the same populations. (C) Variable importance in projection scores is from the PLS-DA model. (D) Univariate analysis of LPC(18:0) (fourth highest VIP score) in control and gemcitabine-dosed cells. *n* = 14, RSD = 33.0% and *n* = 15, RSD = 38.8%, respectively.

Figure 3A,B demonstrates the separation of the control PANC-1 cell group from the gemcitabine-dosed cells when analyzed by principal components analysis (PCA) and partial least-squares discriminant analysis (PLS-DA), respectively. Both groups of cells separate without any overlap of the 95% confidence ellipses. The lipids responsible for the greatest separation in PLS-DA are shown in the order of their variable in projection (VIP) score in Figure 3C, with the greatest contributor being LPC(16:0). The PLS-DA model was evaluated with leave-one-out cross-validation, the results of which are shown in Table S5.

We clearly demonstrate that changes in the lipidome of PANC-1 cells in response to chemotherapeutic treatment can be differentiated at the single-cell level using this methodology. Cross-validation of the PLS-DA model (Table S5) suggests that this difference is indeed due to the measured lipids and not over modeling. The difference between Q2Y and R2Y for two components is less than 0.3, indicating a low level of irrelevant parameters/outliers, and a *Q*2 value of ∼0.7 suggests good predictive accuracy.^46^

Two isomers, LPC(0:0/16:0) and LPC(16:0/0:0), had the first and second-highest VIP scores in the PLS-DA model. Confident identification was based on fragmentation data in bulk analysis. Specifically, the sn1 isomer can be distinguished from the sn2 by a later retention time (Figure S9) and a fragment of *m*/*z* 104 in addition to the PC headgroup fragment of *m*/*z* 184 (Figure S10).^47^ It would not have been possible to distinguish the two isomers in single cells if not for retention time matching of LC-MS data. LPC(0:0/16:0) is observed in 13/14 control cells but 0 gemcitabine-treated cells. LPC(18:0) is also significantly decreased (*p* < 0.001) in the gemcitabine-treated cells (Figure 3D). A decrease of LPCs after gemcitabine treatment has been previously observed in bulk cell analysis and has been linked with drug resistance.^48^ This demonstrates the ability of this single-cell lipidomics workflow to observe changes in cellular lipids, consistent with expected outcomes. These trends in LPCs could indicate that the gemcitabine-treated cells have survived due to the formation of lipid droplets, which are heavily implicated in chemoresistance to gemcitabine.^49−51^

This technique opens up the opportunity to measure lipids in single cells using the retention time and accurate mass to give confidence in the class assignment. The mass spectrometry analysis is applicable not only to capillary sampling but also opens up the opportunity to study lipids in cells isolated using other approaches.

## Conclusions

This work has demonstrated the first example of nanocapillary sampling coupled with analytical flow LC-MS for single-cell lipidomics. Furthermore, the success of this methodology has been demonstrated in its ability to (A) distinguish chemotherapeutically treated PANC-1 cells from controls based on their lipidome and (B) make lipidomic observations consistent with previous observations in the literature. Finally, this methodology has demonstrated unique capabilities otherwise inaccessible to mass spectrometry imaging as living cells were sampled.

This work represents an encouraging proof of concept for single-cell lipidomics and highlights some significant biological and analytical challenges for future studies to address. Specifically, the improvement of confidence in lipid identifications through direct MS/MS acquisition of single cells is highly desirable and should be a top priority for future single-cell lipidomics work. Second, although this work showed an improvement in the variance with respect to sample transfer, there is still a considerable loss in sample which must be addressed in future work. Reducing sample loss will drastically improve the depth of information and quality of comparison between single cells. Finally, the improvements in sensitivity demonstrated here by improved chromatographic separation clearly show that more of the single-cell lipidome can be accessed with better analyte separation.

## Supporting Information Available

Demonstrations of experimental apparatus, details of the internal standard, chromatographic and mass spectrometry parameters, additional data pertaining to chromatography and ionization probe temperature, example extracted ion chromatograms from single cells, bulk cell extract and blanks, MS/MS data, and cross-validation for multivariate analysis (PDF)
