# Supplementary material for: Single-Cell Lipidomics Using Analytical Flow LC-MS Characterizes the Response to Chemotherapy in Cultured Pancreatic Cancer Cells
Source: Anal Chem. 2023 Sep 19;95(39):14727–35. doi: 10.1021/acs.analchem.3c02854 (PMC10551860; doi:10.1021/acs.analchem.3c02854)
Supplement: Supplementary file 1 — ac3c02854_si_001.pdf [file ac3c02854_si_001.pdf]

Supporting Information

Single Cell Lipidomics Using Analytical Flow LC-MS Characterises The Response to Chemotherapy in Cultured Pancreatic Cancer Cells

Kyle D. G. Saunders[1], Johanna von Gerichten[1], Holly-May Lewis[2], Priyanka Gupta[3], Matt Spick[2], Catia Costa[4], Eirini Velliou[3], Melanie J. Bailey[1*]

[1] Department of Chemistry, University of Surrey, Guildford, UK

[2] Faculty of Health & Medical Sciences, University of Surrey, Guildford, UK

[3] Centre for 3D Models of Health and Disease, University College London - Division of Surgery and Interventional Science, London, UK.

[4] Ion Beam Centre, University of Surrey, Guildford, UK

***Correspondence**:

Corresponding Author:

m.bailey@surrey.ac.uk

Table of Contents

Cover page and table of contents……………………………………………………………………………S1-2

Photographic demonstration of cell sampling and cell transfer………………………………………………..S3

Equation 1 – Cell Transfer Efficiency…………………………………………………………………………S3

Tables S1-3 – Internal standard, chromatographic parameters, mass spectrometry parameters……………….S3-5

Extracted ion chromatogram of PC(15:0/18:1(d7)) and PE(15:0/18:1(d7))………………………………………S6

Ionisation probe temperature findings…………………………………………………………………......S6-7

Extracted ion chromatograms of lipids in single cells, blanks and bulk cell extraction………………………..S7-8

Leave-one-out cross validation results of PLS-DA……………………………………………………………S8

Extracted ion chromatograms and MS/MS spectra of two observed isomers of LPC(16:0)……………………S9

Nanocapillary Sampling

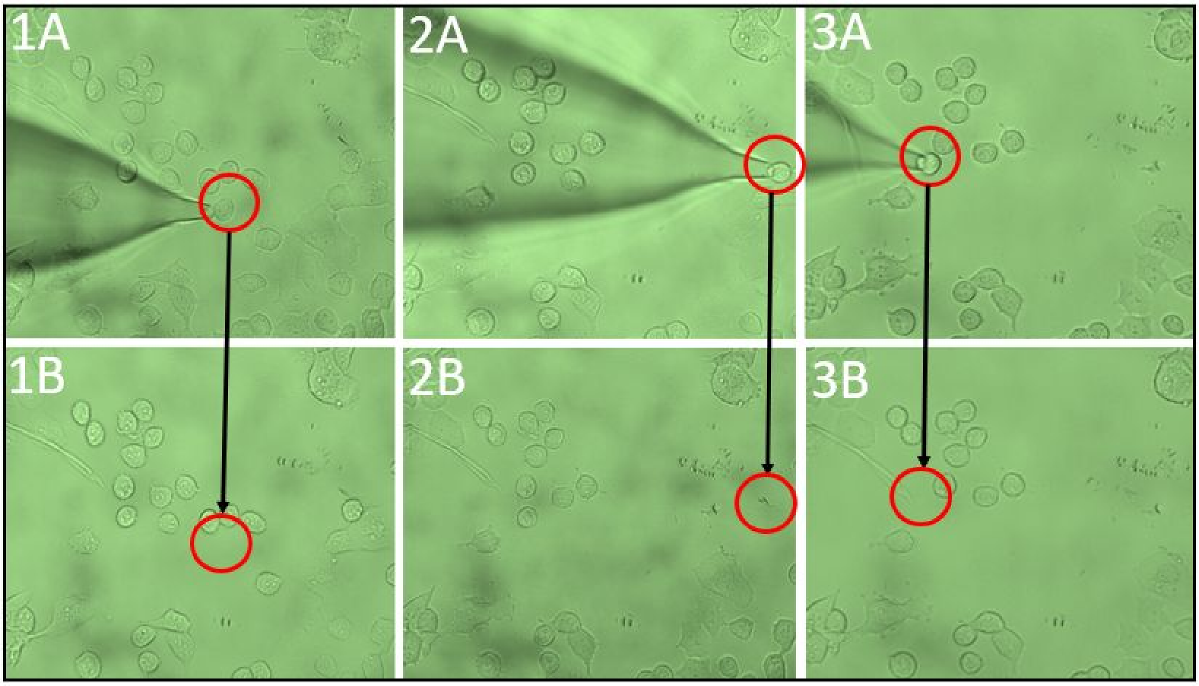

Figure S1 *Nanocapillary sampling allows for isolation of single cells as well as retention of spatial information. PANC-1 cells shown in image, average cell diameter = 15 µm.*

Lipid Transfer

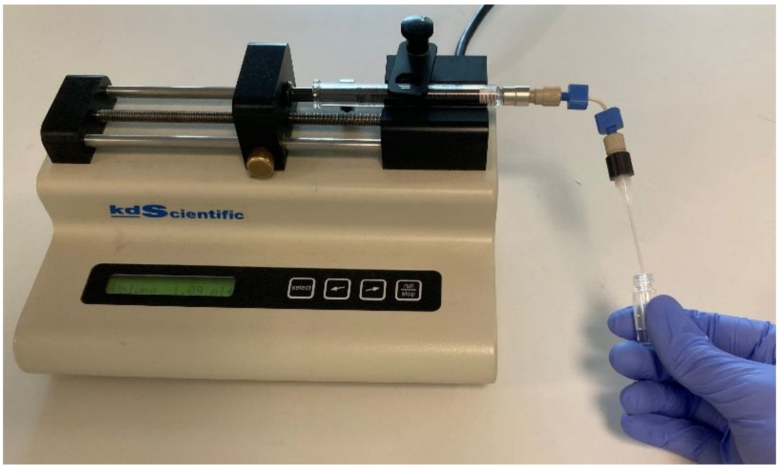

Figure S2 *Single cell sample transfer to LC vial using gas syringe and driver.*

$$Transfer\ Efficiency(\%) = \frac{Average\ lipid\ peak\ area\ (transferred)}{Average\ lipid\ peak\ area\ (control)} * 100$$

Equation S1 *– Transfer efficiency of lipid classes.*

Table S1 *List of deuterated lipid standards in EquiSPLASH®, detectable at 16 ng/mL.*

EquiSPLASH standard	Expected *m/z*	
	**M+H adduct**	**M+NH_4 adduct**
PS (15:0-18:1(d7))	755.5562	
PC (15:0-18:1(d7))	753.6134	

PG (15:0-18:1(d7))		759.5875
SM(d18:1-18:1(d9))	738.6470	
PE (15:0-18:1(d7))	711.5664	
C15 Ceramide (d7)	531.5477	
Lyso PC (18:1(d7))	529.3994	
Lyso PE (18:1(d7))	487.3524	
DAG (15:0-18:1(d7))		605.5844
TAG (15:0-18:1(d7)-15:0)		829.7985

Chromatographic Conditions

Table S2 *Liquid chromatography parameters assessed for lipid coverage.*

<table>
<tr><th>Parameter</th><th>C18 Method</th><th>C30 Method</th></tr>
<tr><td>Column</td><td>Waters Acquity BEH C18 (1.7 μm, 2.1 x 100 mm)</td><td>Thermo Accucore C30 (2.6 μm, 2.1 x 150 mm)</td></tr>
<tr><td>Solvent A</td><td>60:40 (ACN/H_2O) + 0.1 % formic acid</td><td>60:40 (ACN/H_2O) + 0.1 % formic acid + 10 mM ammonium formate</td></tr>
<tr><td>Solvent B</td><td>90:10 (IPA/ACN) + 0.1 % formic acid</td><td>85:10:5 (IPA/H_2O/ACN) + 0.1 % formic acid + 10 mM ammonium formate</td></tr>
<tr><td>Temperature</td><td>55 °C</td><td>40 °C</td></tr>
<tr><td>Injection Volume (μL)</td><td>5</td><td>5</td></tr>
<tr><td>Flow Rate (mL/min)</td><td>0.30</td><td>0.35</td></tr>
<tr><td>Gradient</td><td>
<table>
<tr><th>Time (min)</th><th>% A</th><th>% B</th></tr>
<tr><td>0.0</td><td>60</td><td>40</td></tr>
<tr><td>1.0</td><td>50</td><td>50</td></tr>
<tr><td>3.6</td><td>31</td><td>69</td></tr>
<tr><td>12.0</td><td>12</td><td>88</td></tr>
<tr><td>14.0</td><td>60</td><td>40</td></tr>
<tr><td>16.0</td><td>60</td><td>40</td></tr>
</table>
</td><td>
<table>
<tr><th>Time (mins)</th><th>% A</th><th>% B</th></tr>
<tr><td>0.0</td><td>70</td><td>30</td></tr>
<tr><td>5.0</td><td>70</td><td>30</td></tr>
<tr><td>5.1</td><td>57</td><td>43</td></tr>
<tr><td>14</td><td>30</td><td>70</td></tr>
<tr><td>14.1</td><td>30</td><td>70</td></tr>
<tr><td>21</td><td>1</td><td>99</td></tr>
<tr><td>24</td><td>1</td><td>99</td></tr>
<tr><td>24.1</td><td>70</td><td>30</td></tr>
<tr><td>28</td><td>70</td><td>30</td></tr>
</table>
</td></tr>
</table>

Mass Spectrometry Parameters

Table S3 *Mass spectrometry parameters used for the Full-MS/DDA method.*

Parameters	*Full MS*	*dd-MS^2*
Resolution	70,000	17,500
AGC Target	1e^6	5e^5
Maximum Injection Time (ms)	400	50
Scan Range (m/z)	200 – 1200	200-1200
Loop Count		10
Isolation Window (m/z)		4.0

Collision Energy (normalised to m/z 500, z =1)	30
Minimum AGC Target (for MS/MS triggering)	2.00 e^3
Dynamic Exclusion Window (s)	6

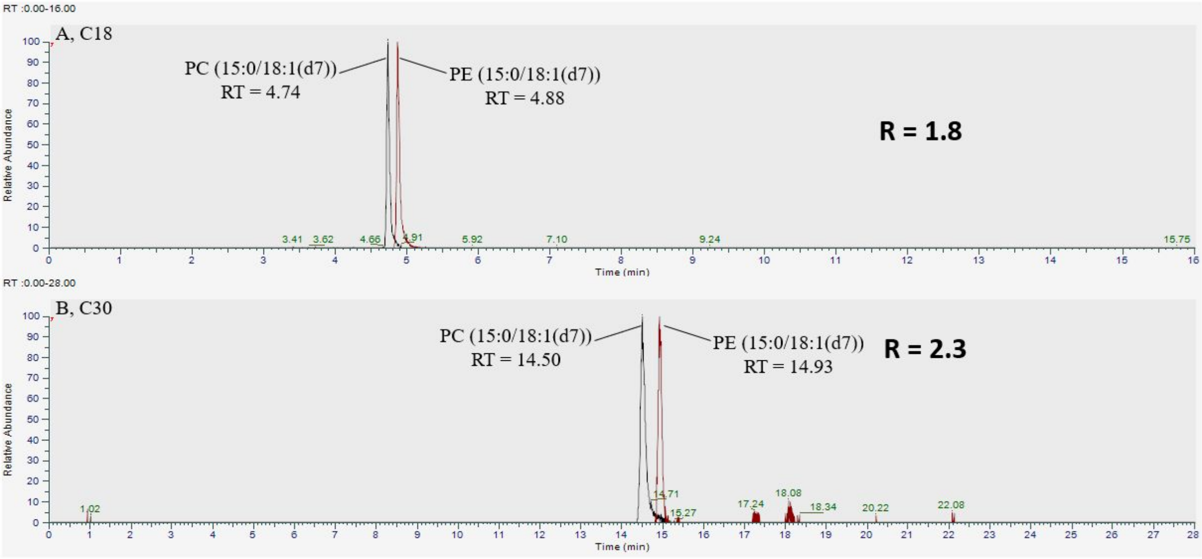

***Figure S3A** and **B** Extracted ion chromatograms of PC(15:0/18:1(d7)) and PE(15:0/18:1(d7)) lipid separated by a **A**.) 16 minute C18-based gradient (resolution =1.8) and, **B**.) 28 minute C30-based gradient (resolution = 2.3).*

Ionisation Probe Temperature

The optimal temperature for lipid ionisation varies significantly according to lipid class. Signal intensity for a PC lipid and TAG lipid in the standard mix are shown in **Figures X and Y**. The optimal temperature for each lipid assessed in shown in **Table Y**.

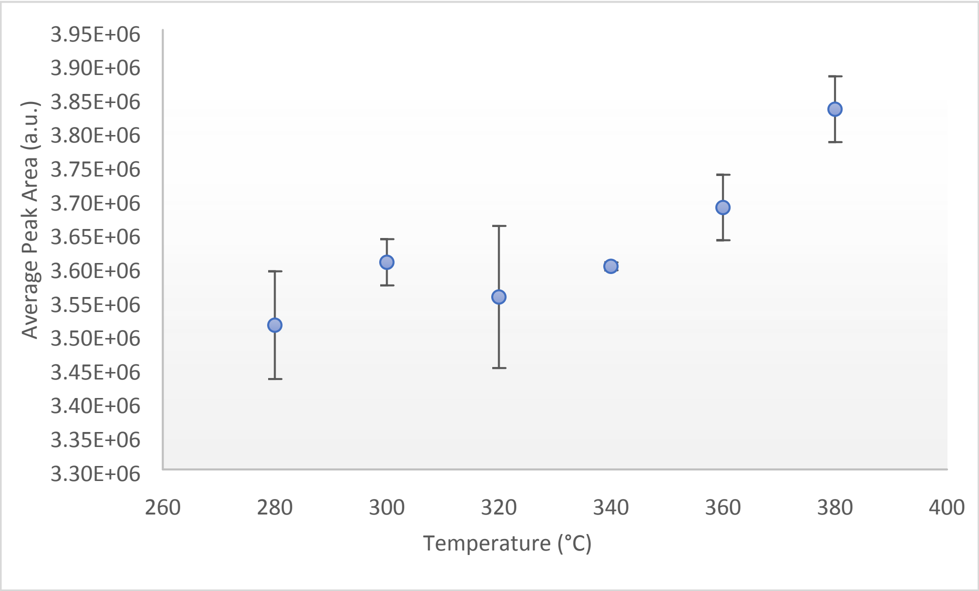

***Figure S4** Average peak area of PC(15:0_18:1(d7)) in response to capillary temperature.*

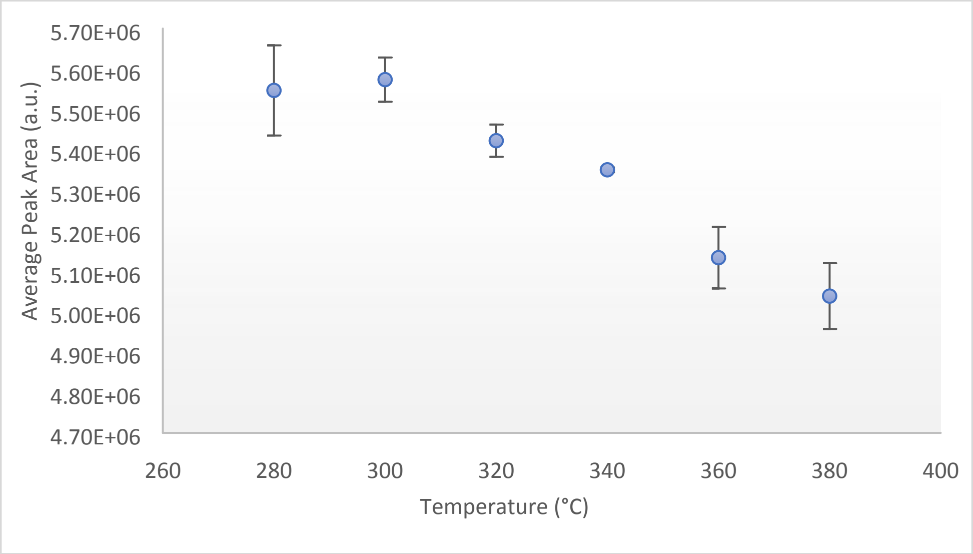

Figure S5 *Average peak area of TAG (15:0-18:1(d7)-15:0) in response to capillary temperature.*

Table S4 *Optimal capillary temperatures of each lipid standard classes.*

	PC	*LysoPC*	*PE*	*LysoPE*	*SM*	*Ceramide*	*PG*	*TAG*	*DAG*
Optimal Temperature (°C)	380	360	300	280	380	360	280	300	280

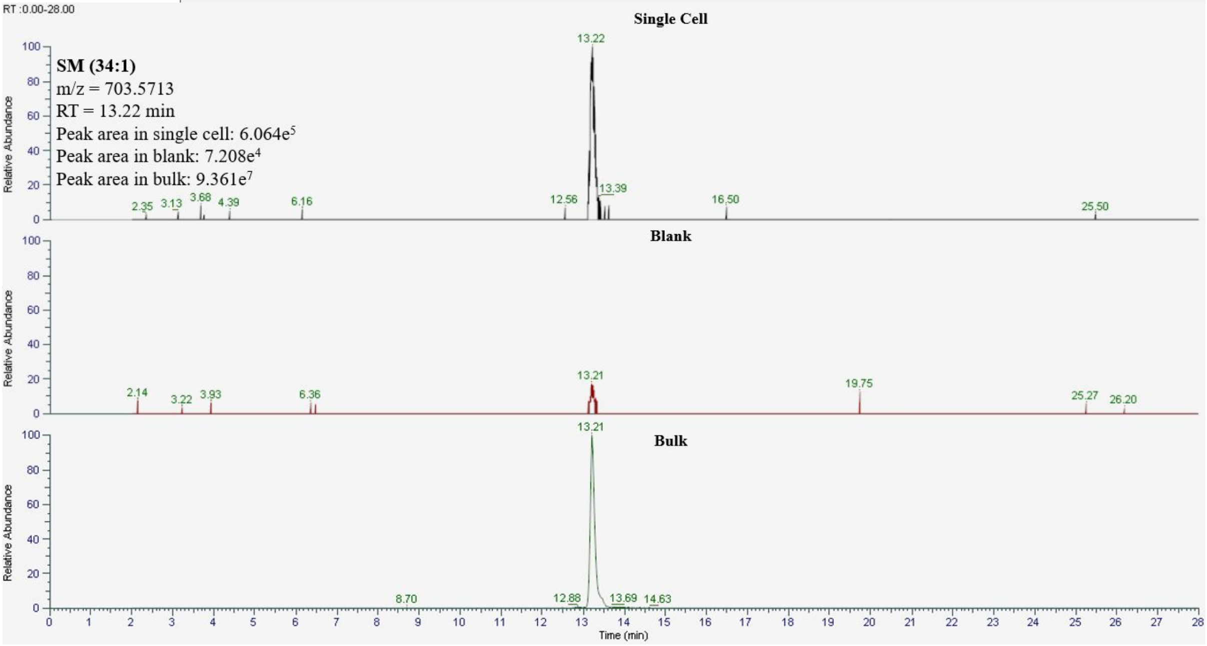

Figure S6 Extracted ion chromatogram of feature assigned to SM(34:1) in a single cell sample vs blank vs bulk.

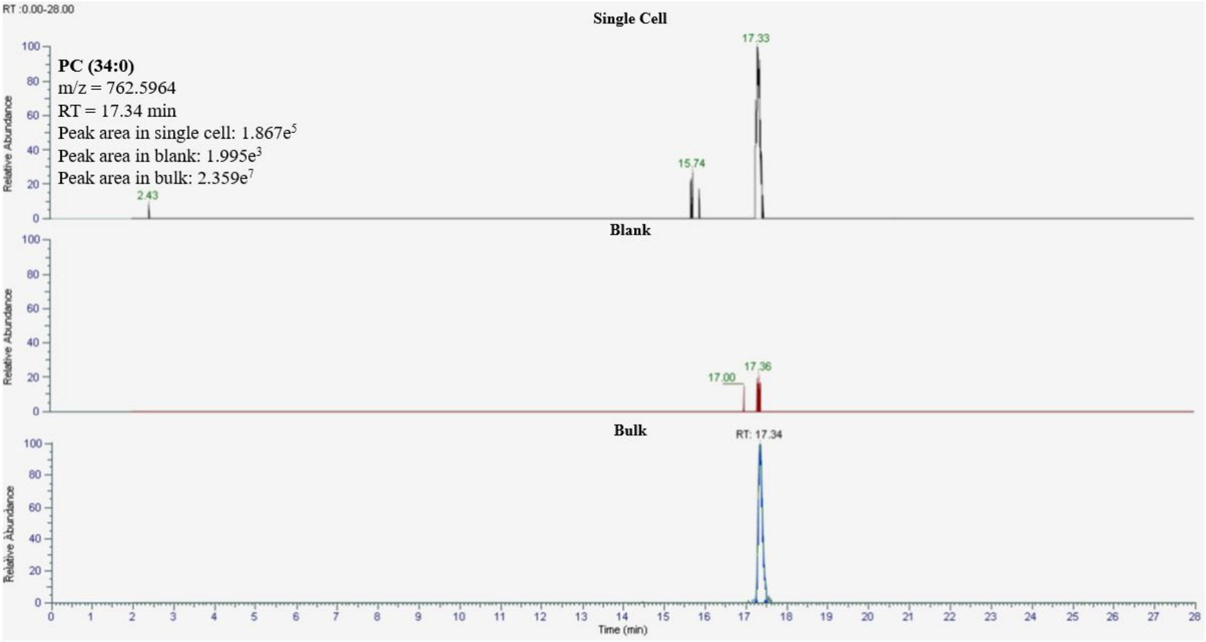

Figure S7 Extracted ion chromatogram of feature assigned to PC(34:0) in a single cell sample vs blank vs bulk.

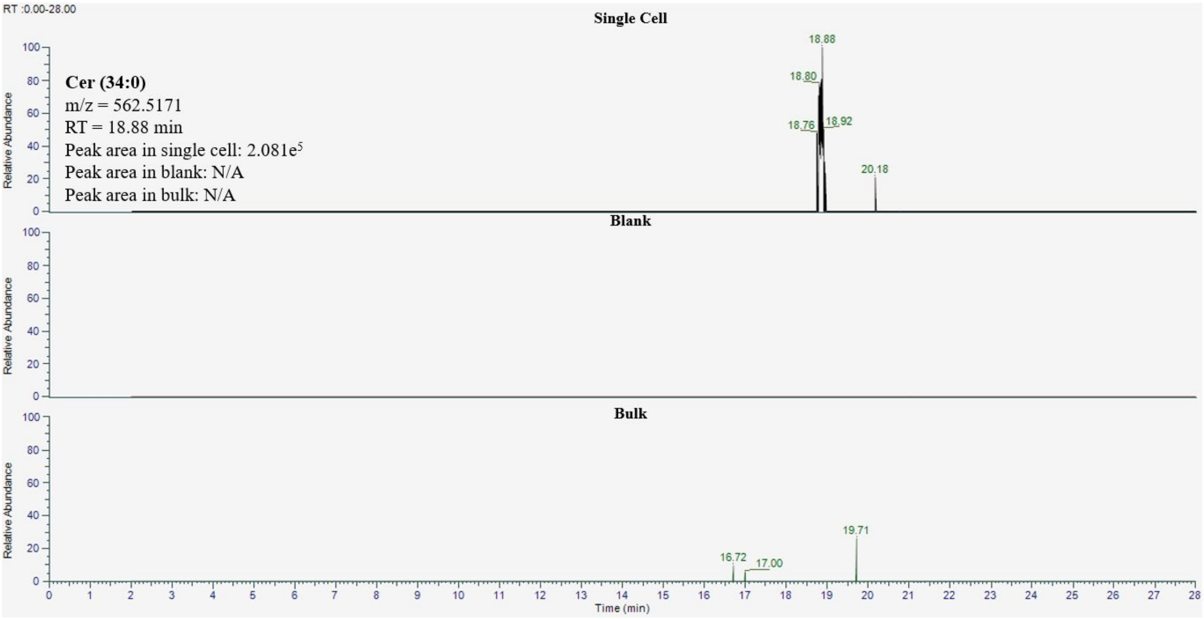

Figure S8 Extracted ion chromatogram of feature assigned to Cer(34:0) in a single cell sample vs blank vs bulk.

Table S5 *Leave one out cross validation results for the PLS-DA model shown in* ***Figure 3B****.*

Measure	1 Component	2 Components	3 Components	4 Components	5 Components
**Accuracy**	0.97	1.0	0.97	0.97	0.93
**R2**	0.88	0.92	0.95	0.97	0.98
**Q2**	0.76	0.84	0.85	0.81	0.76

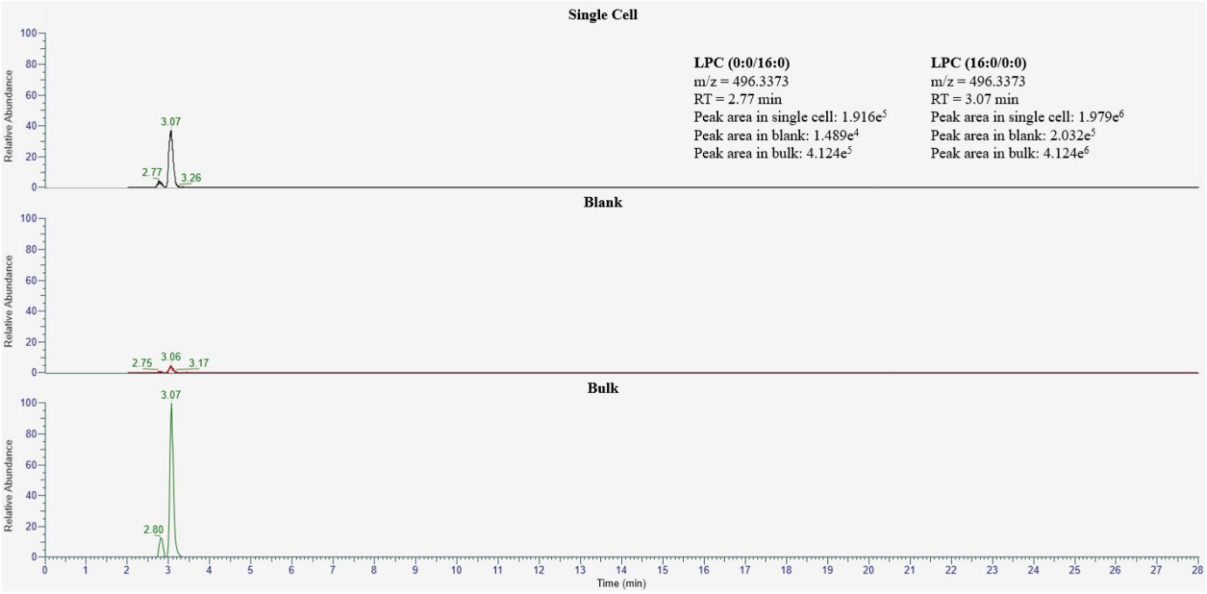

Figure S9 *Extracted ion chromatogram for the two isomers of LPC(16:0) in a single cell, blank and bulk injection.*

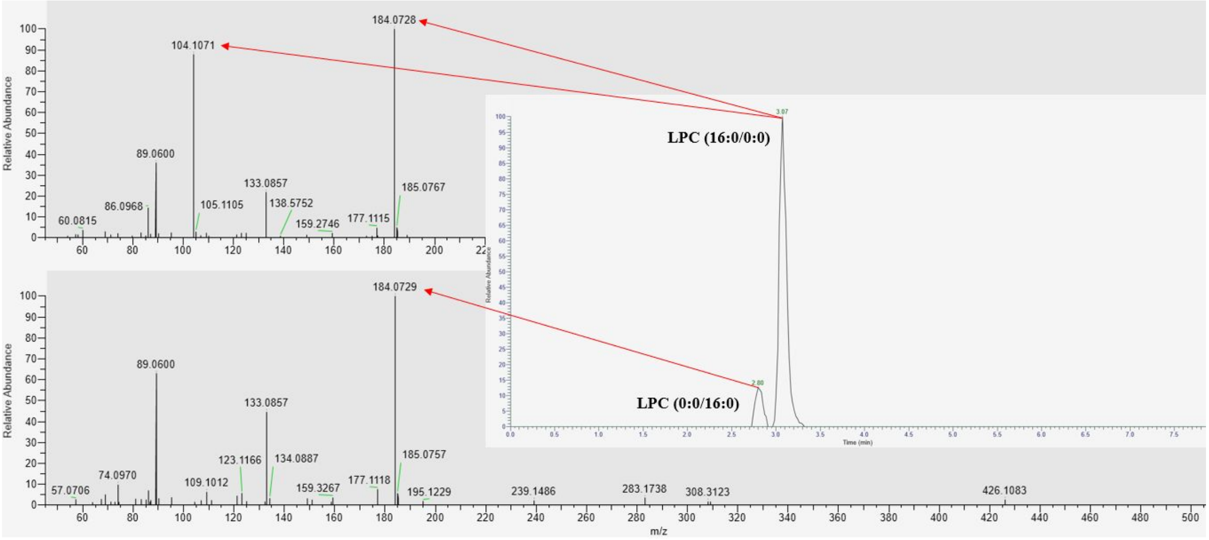

Figure S10 *Confirmatory MS/MS fragmentation spectra of the two isomers of LPC(16:0).*
